# Gastric teratoma presenting as gastrointestinal bleeding in a neonate: a case report and review of literature

**DOI:** 10.1093/jscr/rjag969

**Published:** 2026-10-27

**Authors:** Deekshya Devkota, Suyog Simkhada, Sukriti Shrestha, Suman B Adhikari, Bal M Basnet, Dinesh Bade

**Affiliations:** Department of Pediatric Surgery, Kanti Children's Hospital, Maharajgunj, Kathmandu 44600, Nepal; Department of Pediatric Surgery, Kanti Children's Hospital, Maharajgunj, Kathmandu 44600, Nepal; Department of Pediatric Surgery, Kanti Children's Hospital, Maharajgunj, Kathmandu 44600, Nepal; Department of Pediatric Surgery, Kanti Children's Hospital, Maharajgunj, Kathmandu 44600, Nepal; Department of Pediatric Surgery, Kanti Children's Hospital, Maharajgunj, Kathmandu 44600, Nepal; Department of Pediatric Surgery, Kanti Children's Hospital, Maharajgunj, Kathmandu 44600, Nepal

**Keywords:** case report, gastric teratoma, neonate, melena, gastrointestinal bleeding, pediatric surgery

## Abstract

Gastric teratoma accounts for ˂1% of pediatric teratomas and typically presents as an abdominal mass, distension, or vomiting, making gastrointestinal bleeding an unusual presentation. A 6-week-old male infant presented with black tarry stools since birth, non-bilious vomiting, and severe anemia requiring four packed red blood cell transfusions before referral. Examination revealed a large supraumbilical mass, and contrast-enhanced computed tomography showed a heterogeneous solid-cystic lesion with calcification abutting the greater curvature of the stomach, suggesting a gastric teratoma preoperatively. During laparotomy, an exophytic intragastric mass was excised with primary gastric repair. Histopathology confirmed a mature teratoma. Melena resolved by postoperative day five, and the infant was well at 1-month follow-up. This case adds to the limited literature on gastric teratoma presenting with gastrointestinal bleeding rather than a mass, and reinforces complete surgical excision as curative.

## Introduction

Gastric teratoma is an extremely rare extragonadal germ cell tumor in children, accounting for ˂1% of all pediatric teratomas [1, 2]. It occurs predominantly in neonates and infants, with most reported cases presenting within the first year of life with a marked male predominance [3, 4]. Most gastric teratomas arise from the greater curvature and show exophytic growth, which explains the usual presentation as an upper abdominal mass, abdominal distension, or vomiting [1, 3].

Melena or hematemesis is uncommon and is usually associated with an endogastric or mixed growth pattern causing mucosal ulceration or intraluminal bleeding [1, 5]. This makes neonatal mature gastric teratoma presenting with persistent melena and anemia requiring transfusion an unusual clinical scenario.

The present case adds to the limited literature on neonatal gastric teratoma presenting with gastrointestinal bleeding and highlights the central role of complete surgical excision in achieving a cure. This case report adheres to the Surgical CAse REports (SCARE) guidelines [6] and presents the patient’s clinical presentation, investigations, and management.

## Case presentation

A 6-week-old male infant was referred to our institution with a history of passage of black tarry stools since birth. It was associated with two episodes of non-bilious, non-projectile vomiting, and a 4-day history of fever. Before referral to our center, the infant had required four packed red blood cell (PRBC) transfusions for severe anemia.

The infant was hemodynamically stable with oxygen saturation maintained in room air; however, pallor was noted over the conjunctiva and oral mucosa on general examination. Abdominal examination revealed a firm, mobile, non-pulsatile mass measuring 10 × 7 cm in the supraumbilical region, spanning the right hypochondrium, epigastrium, and left hypochondrium medially. The mass had well-defined, regular margins, and a smooth surface.

Laboratory workup at admission revealed a marked leukocytosis (WBC 17010/cumm; reference 4000–11 000/cumm) with a normal neutrophil percentage, along with a normocytic normochromic anemia (Hb 7.8 g/dl; reference 11.5–18 g/dl) and a normal coagulation profile. C-reactive protein was 187.9 mg/L (reference <5 mg/L) and serum alpha-fetoprotein (AFP) was markedly elevated at 128 IU/ml (reference <6.5 IU/ml). Other laboratory parameters were within normal limits.

Contrast-enhanced computed tomography (CECT) of the abdomen and pelvis demonstrated a large heterogeneous solid-cystic lesion in the central and left upper abdomen, measuring 9.4 × 8.1 × 5.4 cm. The lesion exhibited mild enhancement of its cystic components with multiple foci of calcification. It was noted to abut the greater curvature of the stomach, left lobe of the liver, and spleen, with associated displacement of adjacent bowel loops. These features were suggestive of a gastric teratoma (Fig. 1).

**Figure 1:**
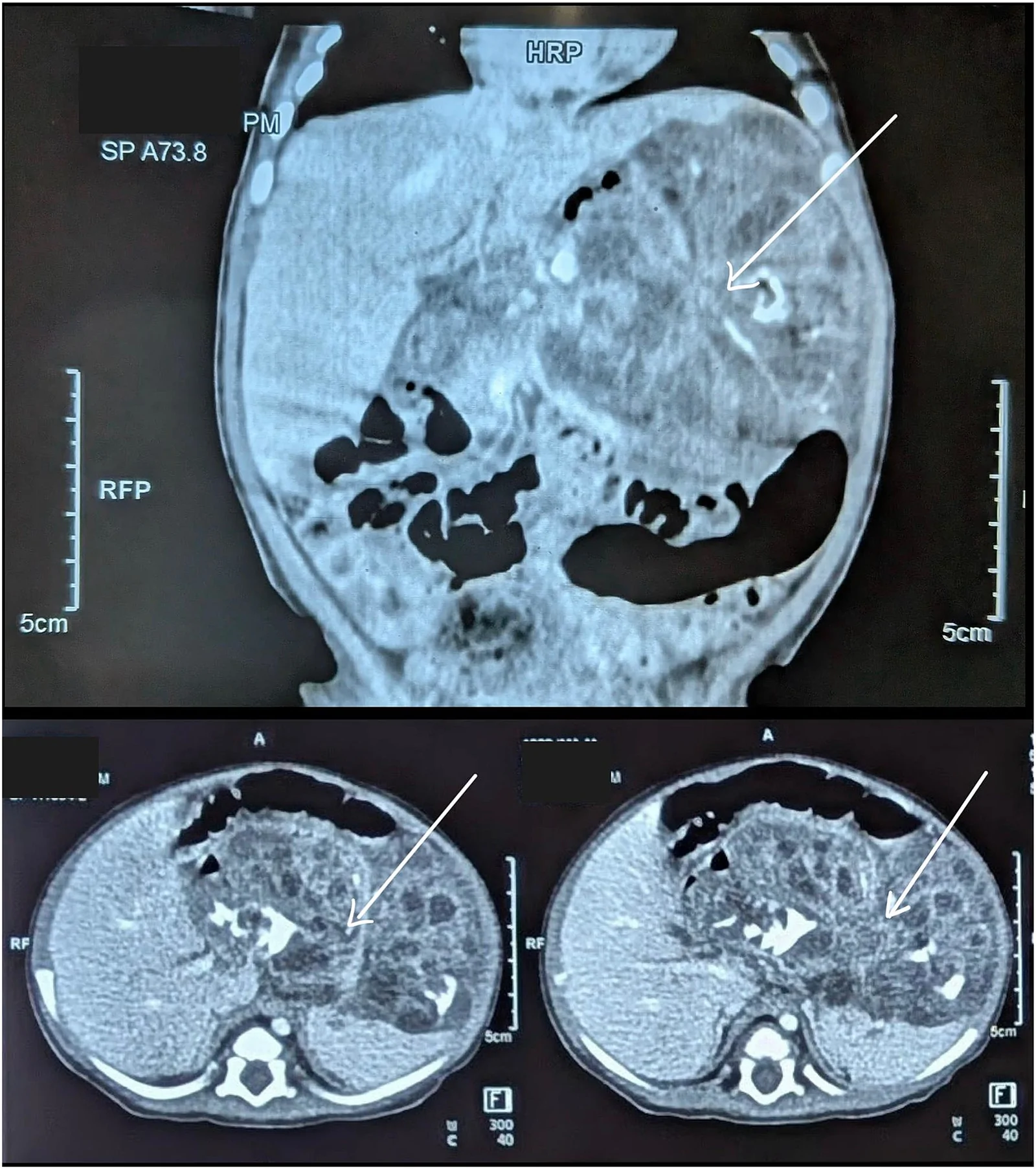
CECT abdomen and pelvis showing a large heterogeneous solid-cystic lesion in the central and left upper abdomen abutting the greater curvature of the stomach, the left lobe of liver, and the spleen (arrows).

Patient underwent an exploratory laparotomy with excision of an intragastric teratoma with primary repair of the stomach. A large, multilobulated, exophytic, solid-cystic mass was found along the fundus and greater curvature of the stomach occupying both inner and outer part of the stomach (Fig. 2). No gross tumor deposits were found in the abdomen.

**Figure 2:**
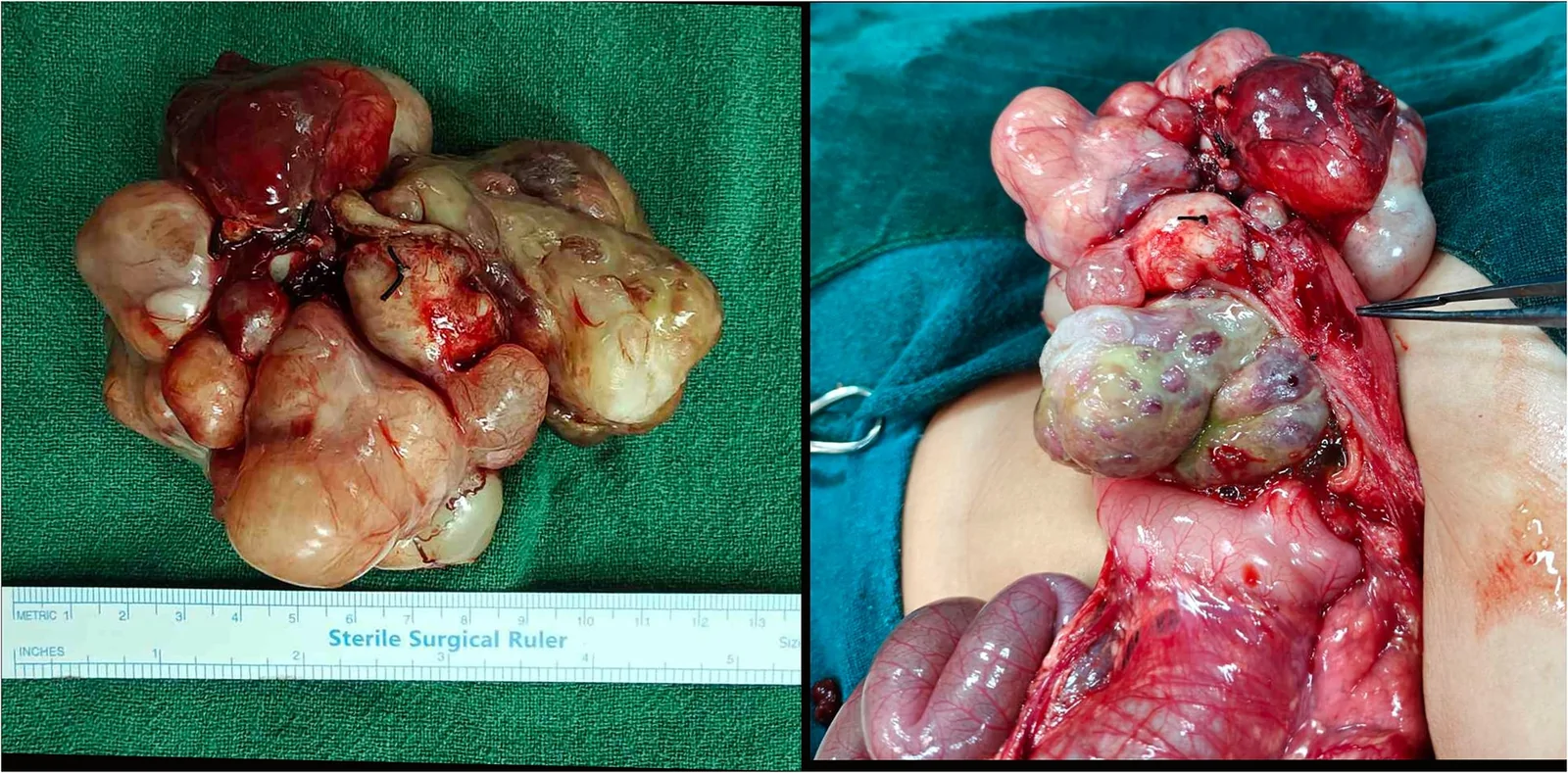
Intraoperative finding showing a large multilobulated, exophytic, solid-cystic mass.

The patient stopped passing black tarry stools by the 5th postoperative day. Ultrasound of the abdomen and pelvis was unremarkable and serum AFP was within normal limits. Histopathological examination revealed mature teratoma composed of well-differentiated tissues without immature or malignant components. The patient’s recovery was uneventful, and he was discharged on postoperative day 10. At 1 and 2 months follow-up, he was feeding well with no recurrence of melena.

## Discussion

Gastric teratoma in neonates and infants usually presents with an upper abdominal mass, abdominal distension, vomiting, or feeding difficulty, while overt gastrointestinal bleeding is described in only a minority of cases [1, 7]. The presentation with melena and severe anemia requiring transfusion, rather than abdominal distension or a mass, is a distinctly rare presentation [3, 8].

Hematemesis or melena usually reflects endogastric or mixed growth with mucosal involvement or ulceration, even when the bulk of the tumor remains exophytic [5]. This mechanism explains the early black tarry stools in our patient and the rapid cessation of bleeding after surgery. Deswal *et al*. [5] described a similar case of a 7-month-old infant with mature endogastric teratoma who initially presented with melena, hematemesis, and anemia requiring transfusions. The patient underwent wide local excision and had a good surgical outcome.

Preoperative diagnosis is difficult because gastric teratoma can mimic more common neonatal abdominal masses like lymphangioma, neuroblastoma, or various tumors of the retroperitoneum [1, 3]. Even so, imaging provides important clues like a heterogeneous solid-cystic mass with calcification [9]. The CECT findings in our case fit this pattern and correctly suspected gastric teratoma before surgery, which is significant because accurate preoperative identification is often challenging.

Published case reports describe excision of the mass together with a portion of the attached gastric wall, followed by primary repair, rather than gastrectomy in every case [10, 11]. This is consistent with the management in our patient, where complete excision with primary gastric repair was sufficient despite the large size of the lesion. Recurrence is mainly due to incomplete resection, which signifies the importance of en bloc tumor removal and careful handling of the gastric attachment [5, 12]. The mature histology in our case, together with complete excision and early clinical resolution of bleeding, therefore supports an excellent short-term prognosis.

Histopathology remains definitive for diagnosis, especially because imaging cannot reliably distinguish mature from immature lesions or exclude focal malignant elements [1, 13]. AFP can be helpful during follow-up, but its value in neonates is limited by physiologically high age-related levels and by the fact that elevated AFP does not always indicate malignant yolk sac differentiation [1, 14]. For that reason, postoperative surveillance is generally done by a combination of clinical review and imaging rather than AFP alone [1, 15].

Overall, our case adds to the small body of literature showing that neonatal gastric teratoma can present primarily as gastrointestinal bleeding with life-threatening anemia rather than as a simple abdominal mass. The main surgical message is that prompt resuscitation, accurate anatomical assessment, and complete excision with primary gastric repair can control hemorrhage rapidly while preserving the stomach. Lack of long term follow up remains the major limitation of this case report.

## Conclusion

Neonatal gastric teratoma can be a possible differential diagnosis when a patient presents with melena or severe anemia along with an upper abdominal mass, especially when imaging shows a mixed cystic-solid lesion with calcification or fat near the stomach. Complete surgical excision with clear margins and gastric preservation when feasible provides a favorable outcome in most reported cases.

## Highlights

- Neonatal gastric teratoma classically presents as an abdominal mass or vomiting, but our infant presented with melena and severe anemia requiring transfusions which is a rare presentation.
- Contrast-enhanced CT correctly suggested gastric teratoma preoperatively by identifying a heterogeneous solid-cystic mass with calcification abutting the gastric greater curvature, aiding diagnosis despite overlap with other neonatal abdominal tumors.
- Bleeding in gastric teratoma suggests endogastric or mixed tumor growth causing mucosal ulceration, explaining the melena in this case and its rapid resolution after excision of the intragastric mass with primary gastric repair.
- Complete surgical excision with gastric preservation was curative, with histopathology confirming mature histology, and no features of recurrence at 1 and 2-month follow-up.

## Supplementary Material

Supplementary_material_rjag969
